# Clinical study on early warning signal monitoring (salivary indicators) of exercise-induced fatigue in technical winter sports athletes

**DOI:** 10.12669/pjms.41.9.11305

**Published:** 2025-09

**Authors:** Yun Fu, Yaming Yu, Weichao Chen

**Affiliations:** 1 Yun Fu Health Care Department, Sichuan Province Orthopedic Hospital, Chengdu, 610041, Sichuan, China; 2 Yaming Yu Department of Sports Medicine, Sichuan Province Orthopedic Hospital, Chengdu 610041, Sichuan, China; 3 Weichao Chen Department of System, Chengdu Gauss Intelligent Electronic Technology Co., Chengdu 610036, Sichuan, China

**Keywords:** Technical sports events, Winter Olympic Games, Exercise-induced fatigue, Signals, Monitoring

## Abstract

**Objective:**

To explore the early warning signals for exercise-induced fatigue in athletes of this category in China by analyzing the values and change rates of fatigue index, with monitoring indicators as the main focus.

**Methodology:**

This was a retrospective study. In 175 Chinese winter athletes in technical winter events such as figure skating, snowboarding big air and slopestyle, ski jumping, and Nordic combined between February 2023 to December 2023 were selected, including 85 males and 90 females, to monitor the values or change rates of early warning indicators for exercise-induced fatigue.

**Results:**

In the 175 national team athletes of technical winter events, the level of salivary testosterone was increased and the level of salivary cortisol was decreased in the excellent group compared with those in the ordinary group, with no significant differences in other salivary biomarkers between the two groups(P>0.05); there was a statistically significant difference (p<0.05) in salivary IgA level between the negative and positive athletes classified by “consecutive 3-day Borg index change rate”, with a significantly lower concentration in the positive athletes; there were statistically significant differences in salivary cortisol, T/C ratio, CK, CKMB, IgA, and α-amylase concentrations between the negative and positive athletes classified by “consecutive five and seven day mean Borg index change rate”.

**Conclusion:**

This novel high-sensitivity and accurate saliva testing may capture stable and specific changes in fatigue indicators, and serve as an early warning for the occurrence of exercise-induced fatigue, effectively preventing athletes from overtraining and related sports injuries.

## INTRODUCTION

Sports competitions in China and worldwide have increased in recent years, and it is difficult for athletes to maintain their competitive state and break through their competition scores. Exercise-induced fatigue is often complicated with psychological fatigue, which even endangers the sports career of the athletes.^1^-^3^ In sports science studies, monitoring of exercise-induced fatigue caused by excessive competition intensity and untimely recovery has always been a focus and challenge in the academic community.

The sports events of the 24th Winter Olympic Games can be classified into different categories according to their features in nature such as intelligence and courage, technique, speed, and confrontation. The technical winter sports events include figure skating, ski jumping, freestyle skiing, snowboarding, and Nordic combined. Blood sample collection from athletes daily or weekly is less feasible because the actual training and management of sports teams in China are affected by the training locations and seasons. Studies have found that various biomarkers are positively correlated in saliva and blood.^4^,^5^ The specificity and sensitivity of salivary indicators are favorable, and the procedure is non-invasive, simple and fast, which is highly accepted by athletes and coaches. Therefore, saliva testing is increasingly used as a biomarker to monitor the body.^6^,^7^

In the present study, saliva samples were collected and tested, and the characteristics of technical winter sports events were considered to explore the early warning signals for exercise-induced fatigue in athletes of this category in China by analyzing the values and change rates of fatigue index, with monitoring indicators as the main focus.

## METHODOLOGY

This was a retrospective study. A group of national training team members preparing for the Beijing Winter Olympics in technical winter events were included in this study, and all the preparation team members were participants. A total of 175 Chinese winter athletes (aged ≥15) in technical winter events such as figure skating, snowboarding big air and slopestyle, ski jumping, and Nordic combined between February 2023 to December 2023 were selected. There were 85 males and 90 females with an average of 24.7±4.8 age (range: 17-31 years). Data were collected for at least 28 consecutive days from each athlete, and a total of 4177 person-times were obtained.

### Ethical approval:

The study was approved by the Institutional Ethics Committee of Sichuan Province Orthopedic Hospital (No.:2019-11-13-1; Date: November 13, 2019), and written informed consent was obtained from all participants.

### Inclusion criteria:

- A whole group of national training team members of the technical winter events who prepared and participated in the Beijing Winter Olympics(aged ≥ 15 years).
- Provided informed consent.

### Exclusion criteria:

- Female athletes during their menstrual period.
- Athletes who experienced upper respiratory tract infections, diarrhea, exercise-induced proteinuria, exercise-induced hematuria, rhabdomyolysis, and other unwanted conditions induced by excessive training load as determined by sports medicine physicians during and within seven days after data collection.

Clinical data of the tested indicators were collected according to the actual condition of the sports team. Briefly, baseline values of the athletes were collected in the morning before training (before breakfast), and resting values after training were collected the next morning in the following order: Borg index, morning pulse, and saliva sample collection. After the formal initiation of training, the relevant data were collected accordingly for 28 consecutive days.

### Outcome Measures:

Borg index and morning pulse of athletes were collected daily, and the concentrations of salivary cortisol(C), testosterone(T), creatine kinase(CK), creatine kinase isoenzyme(CKMB), IgA, IgM, and alpha-amylase were determined.^8^

### Statistical Analysis:

The daily change rates of the Borg index and morning pulse relative to the baseline values were calculated using the following formula:


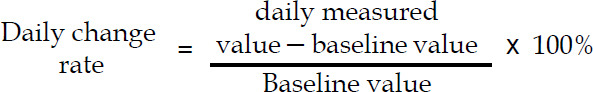


If a baseline value of 0 was reported by the athlete, the baseline value in the change rate calculation was converted to 0.001 because using 0 as a denominator was not acceptable. According to the daily change rate, the mean change rates of the Borg index and morning pulse were calculated for consecutive three days, five days and seven days. For each consecutive seven days, the mean change rate was from Monday to Wednesday: (21 + 22 + 23)/3 and from Tuesday to Thursday: (22 + 23 + 24)/3 for 3 consecutive days; from Monday to Friday: (21 + 22 + 23 + 24 + 25)/5, and from Tuesday to Saturday: (22 + 23 + 24 + 25 + 26)/5 for 5 consecutive days; and from Monday to Sunday: (21 + 22 + 23 + 24 + 25 + 26 + 27)/7 for seven consecutive days.

### Statistical Analysis:

SPSS 21.0 was used for data analysis. Measurement data with normal distribution were presented as mean ± standard deviation, and those without normal distribution were presented as median and interquartile range [M(P_25_ - P_75_)]. The recommended ranges of absolute values and change rates for the Borg index and morning pulse were determined using the guidelines for medical reference calculation. The 95% percentile point was determined. For measurement data with normal distribution, the 95% one-sided upper limit was mean + u0.05*S (S was the standard deviation), and for those without normal distribution, the one-sided upper limit of P_95_ was used.

P_95_ of each tested index was used as the cutoff value. The index was negative if the result was lower than P_95_, and positive if higher than P_95_. Independent sample t test was used for comparisons of salivary biomarkers between groups, and univariate analysis of variance was used to analyze the differences in morning pulse and each biomarker between the negative and positive athlete groups defined by different evaluation criteria. Logistic regression analysis was performed on indicators with a p<0.1 identified in the independent sample t test. Borg index- and morning pulse-related indexes were used, respectively, to determine the fatigue state of athletes. The fatigue state was used as the dependent variable, and indicators with p<0.1 were used as independent variables and introduced into the model using the stepwise backward regression method of maximum likelihood estimation, α=0.05.

## RESULTS

According to the athlete level, these athletes were divided into the excellent group (higher than level-1) and the ordinary group (Grade-I and below). The two groups were compared and analyzed to explore the differences in indicators between athletes of different levels. The general data of athletes in different groups are shown in Table-I and Table-II. The results showed that the level of salivary testosterone was increased and the level of salivary cortisol was decreased in the excellent group compared with those in the ordinary group, with no significant differences in other salivary biomarkers between the two groups (P>0.05) (Table-III).

**Table-I:** General data of male athletes in different groups.

Groups	n	Age	Training years
Ordinary	17	19.44±1.90	8.03±0.74
Excellent	68	26.17±3.91	14.98±3.28

**Table-II:** General data of female athletes in different groups.

Groups	n	Age	Training years
The ordinary group	19	18.92±2.21	7.67±0.80
The excellent group	71	26.81±4.03	15.67±3.26

**Table-III:** Comparison of biomarkers between athletes of different levels.

Salivary biomarkers	The excellent group	The ordinary group	t	p
Testosterone(nmol/L)	0.85±0.23	0.79±0.24	7.362	0.000**
Cortisol(nmol/L)	36.43±9.14	39.56±10.05	-2.397	0.029*
Creatine kinase (ng/mL)	10.79±2.74	10.08±2.83	0.661	0.518
IgA(μg/mL)	4.80±1.33	4.79±1.29	0.162	0.873
IgM(μg/mL)	5.61±1.86	5.66±1.87	-0.556	0.586
Salivary Alpha amylase(U/mL)	86.87±22.81	89.46±19.96	-1.110	0.283

* p<0.05,

** p<0.01

A total of 4177 person-times of Borg and morning pulse data were obtained in this study. The 95% percentile points of the Borg index, absolute value of one-day morning pulse and their change rates defined according to the guidelines for the calculation of 95% medical reference values were shown in Table-IV. In athletes with a negative or positive test of each index, indexes in athletes with statistically significant differences in salivary biomarkers were shown. No statistically significant differences in biomarkers were observed between the negative and positive athletes classified by “one-day Borg change rate”, “one-day morning pulse absolute value”, “1-day morning pulse change rate”, and “consecutive three-day morning pulse change rate” (P>0.05); there was a statistically significant difference (P<0.05) in salivary C concentration between the negative and positive athletes classified by the “one-day Borg index absolute value”, with a significantly higher concentration in the positive athletes; there was a statistically significant difference (P<0.05) in the salivary IgA level between the negative and positive athletes classified by “consecutive three-day mean Borg index change rate”, with a significantly lower level in the positive athletes; there were statistically significant differences in salivary C, T/C ratio, CK, CKMB, IgA, and salivary α-amylase concentrations between the negative and positive athletes classified by “consecutive 5-, and seven-day mean Borg index change rates”; and there were statistically significant differences in the salivary T/C ratio and IgA concentration between the negative and positive athletes classified by the “consecutive 5- and 7-day mean morning pulse change rates”.

**Table-IV:** Biomarkers with differences in 95% intervals in Borg index and morning pulse in different consecutive days.

Indexes	Evaluation criteria	mean±standard deviation	>95%	biomarkers with statistical differences between athletes with negative and positive indexes
Borg Index	1-day Borg index	11.90±1.82	15.00	C
	1-day change rate(%)	14.72±6.73	33.33	/
	Consecutive 3-day mean change rate (%)	11.68±5.71	24.80	IgA
	Consecutive 5-day mean change rate (%)	10.17±5.66	24.58	C, T/C ratio, CK, CKMB, IgA, salivary α-amylase
	Consecutive 7-day mean change rate (%)	9.15±5.43	23.36	C, T/C ratio, CK, CKMB, IgA, salivary α -amylase
Morning pulse	1-day morning pulse	58.79±6.97	70.00	/
	1-day change rate(%)	2.57±1.68	4.29	/
	Consecutive 3-day mean change rate (%)	2.13±1.05	4.00	/
	Consecutive 5-day mean change rate (%)	1.99±0.87	3.97	T/C ratio, IgA
	Consecutive 7-day mean change rate (%)	1.74±0.81	4.01	T/C ratio, IgA

Based on the above results, athlete fatigue determined by the Borg index and related criteria was divided into three categories, namely “1-day Borg index absolute value”, “one-day and consecutive three-day mean Borg index change rate “, and “consecutive 5- and 7-day mean Borg index change rate”. Fatigue state in the same batch of athletes was determined using the three evaluation criteria, and logistic regression was used to construct models of various fatigue states and salivary biomarkers, using the presence of fatigue as the dependent variable, and the indexes with P<0.1 in the table as the independent variables. The independent variables were introduced into the model using the stepwise backward regression method of maximum likelihood estimation. As shown in Table-V, a one-day Borg index absolute value of greater than 15 was positively correlated with increased salivary C, an increased one-day and consecutive 3-day mean Borg index change rate was positively correlated with decreased IgA concentration, and an increased consecutive 5- and 7-day mean Borg index change rate was positively correlated with decreased T/C ratio, decreased IgA concentration, and increased C (P<0.05).

**Table-V:** Logistic multiple regression of Borg index-related indicators.

Evaluation criteria	Indicators	B	P	OR
1-day Borg index absolute value	C	0.041	0.017	1.041
	T/C ratio	-0.221	0.083	0.021
	Constant	-5.151	0.031	0.006
1-day, consecutive 3-day mean Borg index change rate	IgA	-0.003	0.045	0.029
	Constant	27.266	0.089	6.621
Consecutive 5- and 7-day mean Borg index change rate	C	0.095	0.072	1.099
	T	-0.839	0.115	0.432
	T/C ratio	-0.944	0.038	0.389
	IgA	-0.002	0.014	0.998
	CK	0.259	0.013	1.295
	Constant	15.534	0.061	6.575

When only “consecutive five and seven days mean morning pulse change rate” was used as an evaluation index, the differences in non-fatigue-related salivary biomarkers between groups were significant (P<0.1). Therefore, logistic regression analysis was conducted using the presence of fatigue determined by this index as the dependent variable and salivary IgA concentration and T/C ratio as independent variables, and the results are shown in Table-VI.

**Table-VI:** Logistic multiple regression of morning pulse-related indicators.

Biomarkers	B	P	OR
IgA	-0.001	0.006	0.009
T/C	-0.567	0.041	0.034
Constant	-2.815	0.053	0.060

## DISCUSSION

It was found in this study athletes followed a common pattern during the training cycle, i.e., the accumulation of fatigue was associated with decreased testosterone and increased cortisol in saliva, indicating the development of exercise-induced fatigue.^9^-^11^ In terms of salivary cortisol, testosterone, and T/C ratio, the pattern of consecutive five and seven days morning pulse change rates indicated the disordered catabolism and anabolism in the athletes and significantly increased CK concentration, which can warn athletes of possible oxygen deficiency, metabolic product accumulation, excessive consumption of energy substances, and oxidative damage of skeletal muscle fibers and tissues, and suggest that exercise-induced fatigue occurs and athletes are entering this stage.^12^-^15^

Salivary biomarkers can sensitively and stably reflect exercise-induced fatigue, and it is reliable to use saliva testing to monitor the development of exercise-induced fatigue in sports teams.^16^-^18^ Saliva testing is a fast, simple and effective monitoring approach in early warning to avoid the adverse accumulation of exercise-induced fatigue in athletes and prevent the occurrence of sports injuries.^18^ In addition, this testing can reflect the best fitness and tolerance of various functional systems of the body under the state of exercise-induced fatigue, providing a basis for the positive feedback of information between coaches and athletes to improve the quality of sports completion.^15^,^19^-^20^

### Limitations:

However, a small number of samples is the shortcoming of this study. In view of this, more samples should be included in future studies to further validate the findings of this study. It is hoped that the findings of this study can be implemented in all sports events at all levels and promoted in public sports, with adequate responses from integrated application of sports and health.

## CONCLUSIONS

In summary, monitoring of exercise-induced fatigue indexes and their specific changes may timely reflect and warn of the occurrence of early exercise-induced fatigue in athletes of technical winter events. The improvement of the physical ability and performance of athletes are closely related key points of the Science and Technology Olympics.

### Authors’ Contributions:

**YF: C**onceived and designed the study, Literature search, Carried out the studies,

**YY:** Involved in the writing of the manuscript and is responsible for the integrity of the study.

**WC:** Literature search, collected the data and performed the analysis.

All authors have read and approved the final manuscript.
